# Integrating field-based heat tents and cyber-physical system technology to phenotype high night-time temperature impact on winter wheat

**DOI:** 10.1186/s13007-019-0424-x

**Published:** 2019-04-24

**Authors:** Nathan T. Hein, Dan Wagner, Raju Bheemanahalli, David Šebela, Carlos Bustamante, Anuj Chiluwal, Mitchell L. Neilsen, S. V. Krishna Jagadish

**Affiliations:** 1 Department of Agronomy, 2004 Throckmorton Plant Sciences Center, Kansas State University, 1712 Claflin Road, Manhattan, KS 66506-5501 USA; 2 Department of Computer Science, Kansas State University, Manhattan, KS 66506 USA

**Keywords:** Cyber-physical system, Heat stress, Heat tents, High night-time temperature, Raspberry Pi, Wheat

## Abstract

**Background:**

Many agronomic traits have been bred into modern wheat varieties, but wheat (*Triticum aestivum* L.) continues to be vulnerable to heat stress, with high night-time temperature (HNT) stress shown to have large negative impact on yield and quality. Global mean temperature during the day is consistently warming with the minimum night temperature increasing at a much quicker pace. Currently, there is no system or method that allows crop scientists to impose HNT stress at key developmental stages on wheat or crops in general under field conditions, involving diverse genotypes and maintaining a dynamic temperature differential within the tents compared to the outside.

**Results:**

Through implementation of a side roll up and a top ventilation system, heaters, and a custom cyber-physical system using a Raspberry Pi, the heat tents were able to consistently maintain an elevated temperature through the night to differentiate heat stress impact on different genotypes. When the tents were placed in their day-time setting they were able to maintain ambient day-time temperature without having to be removed and replaced on the plots. Data averaged from multiple sensors over three consecutive weeks resulted in a consistent but small temperature difference of 0.25 °C within the tents, indicating even distribution of heat. While targeting a temperature differential of 4 °C, the tents were able to maintain an average differential of 3.2 °C consistently throughout the night-time heat stress period, compared to the outside ambient conditions. The impact of HNT stress was confirmed through a statistically significant yield reduction in eleven of the twelve genotypes tested. The average yield under HNT stress was reduced by 20.3% compared to the controls, with the highest reduction being 41.4% and a lowest reduction of 6.9%. Recommendations for fine-tuning the system are provided.

**Conclusion:**

This methodology is easily accessible and can be widely utilized due to its flexibility and ease of construction. This system can be modified and improved based on some of the recommendations and has the potential to be used across other crops or plants as it is not reliant on access to any hardwired utilities. The method tested will help the crop community to quantify the impact of HNT stress, identify novel donors that induce tolerance to HNT and help the breeders develop crop varieties that are resilient to changing climate.

**Electronic supplementary material:**

The online version of this article (10.1186/s13007-019-0424-x) contains supplementary material, which is available to authorized users.

## Background

Winter wheat (*Triticum aestivum* L.), with centuries of genetic improvement, has acquired a suite of favorable traits essential for adaptation to a wide range of environmental conditions. Some of the key developments in wheat breeding and domestication includes larger grain size and a phenotype without seed shattering [1]. Further improvements benefitting from technological advances over the last century by introducing high yielding varieties, fertilizer, pesticides, and modern equipment, have resulted in translating wheat into one of the major staple cereals of the world. Over the last six decades (1961 and 2016) the overall production of wheat has increased by over 500 million tonnes with only a 15.9 million ha increase in harvested area [2]. Improved genetic and management interventions have transformed the average wheat yield from 1.09 t ha^−1^ in 1961 to 3.41 t ha^−1^ in 2016 [2]. In spite of the dramatic increase in overall wheat production, the rate of increase in production is unable to meet the current or the predicted global demand for the future [3]. Even though the annual per capita consumption of wheat is expected to drop by about one percent, the overall annual consumption of wheat is predicted to increase by almost 90 Mt between 2014 and 2024, as a result of increasing population and demand from the biofuel industry [4].

The two main components determining wheat yield potential are the number of grains per meter square and the average weight of each grain [5]. Many genetic, environmental, and field management decisions can alter physiological processes that determine grain number and weight and eventually grain yield. Some of these factors include nutrient availability, temperature, water and solar radiation, fertilizer, and genotype [6]. Among the environmental factors, high temperatures during flowering and grain filling have shown to induce significant loss in grain numbers and weight [7, 8]. Although the overall average temperature has warmed across the globe, recent analysis has shown that the daily minimum temperature (occurring during the night) is increasing at a faster rate than the daily maximum temperature [9, 10]. Hence, it is important and timely to understand the impact of high night-time temperature (HNT) on crops in general and in the sensitive field crops including winter wheat.

During 1979 and 2003, the annual mean maximum temperature increased by 0.35 °C and the annual mean minimum temperature increased by 1.13 °C at the International Rice Research Institute experimental farm, Philippines. As a result, the rice yield decreased by 10% for every 1 °C temperature increase in mean minimum temperature during the dry season [11]. The same study found that the increase in mean maximum temperature did not have the same effect on yield as the mean minimum temperature [11]. Recent studies on the effects of HNT stress on different field grown crops has, until now used (i) field-based tents with a static system [12–15] or (ii) much smaller tents with a cyber-physical system that captures single genotype responses to HNT stress and has to be physically placed and removed daily [16]. The impact of HNT and the physiological route through which yield and quality losses occur has been documented in rice using field-based heat tents [12–14, 17]. Although the existing field tents at IRRI, Philippines, can potentially include moderate number of genotypes, the HNT treatment imposition is static at a predetermined target temperature while the outside temperature can vary quite dynamically. A cyber-physical system is a computer system that incorporates electrical engineering and computer science to bridge the digital and physical worlds through the use of embedded technology [18]. Through the use of software and sensors, the cyber-physical system is able to interact with and react to their environment. The only field experiment involving wheat, HNT, and a cyber-physical system used 3 m × 1.3 m × 1.3 m structures that were manually placed on plots of a single variety of wheat called Baguette 13 for 12 h every night from the third detectable stem node to 10 days post-flowering. This experiment recorded a 7% reduction in grain yield along with a reduction in biomass and grain number [16].

Phenotyping facilities such as rain-out shelters for quantifying drought stress responses [19, 20] and the use of naturally occurring hotter summer conditions have been extensively used to study the impact of high day-time temperature (HDT) stress across crops [21–23]. However, there doesn’t exist a large field-based phenotyping system that can capture larger genetic diversity for HNT responses at critical growth and developmental stages and at the same time induce a dynamic HNT treatment closely following the outside ambient temperature. Hence, our major objective was to develop and test a robust field-based cyber-physical system by modifying a currently available HDT stress heat tent. The overall aim was to impose a HNT stress of 4 °C automatically following the dynamic changes in the open field i.e., outside the structures and simultaneously capturing genetic diversity for HNT stress impact on physiological parameters and grain yield. While the system and methodology developed is tested on winter wheat, there is potential that this technology is scalable and can be extended to crops or plants of interest to the scientific community, although this is yet to be evaluated.

## Materials and methods

### Heat tent

The heat tents that were used for this specific project were built and used in previous studies to quantify HDT effects on wheat and sorghum [8, 24, 25]. Each tent was built using a steel frame for the base and heavy piping to create the sidewalls and apex. The heat tents were constructed in the Gothic style with vertical framing every 1.2 m along the sidewall. The heat tents are 7.2 m long, 5.4 m wide, and 3.0 m tall at the apex. Lock channel and wiggle wire was installed around the available edges of the frame to enclose the tent. The heat tents were enclosed using polyethylene film (6 mil Sun Master^®^ Pull and Cut Greenhouse Film) with 92% light transmission according to the manufacturer. New plastic was installed on all the tents before the start of the experiment. The main components in converting the HDT tents into HNT included the top vent, side roll vents, heating system, and a cyber-physical thermostat controller system operated by a Raspberry Pi.

#### Top vent

In order to maintain ambient conditions throughout the day within the tents, the top vent (Fig. 1.1) was kept functional from the HDT set up. In previous experiments, the top vent was used to prevent excess heating above a set temperature by opening the vent when the desired temperature target was met. However, in the HNT set up, the top vent was opened throughout the day to maintain temperature within the tent closer to ambient conditions to prevent confounding our HNT research by imposing HDT stress. The vent was forced closed during the night to impose and maintain a consistent level of elevated temperature compared to the outside ambient temperature.

**Fig. 1:**
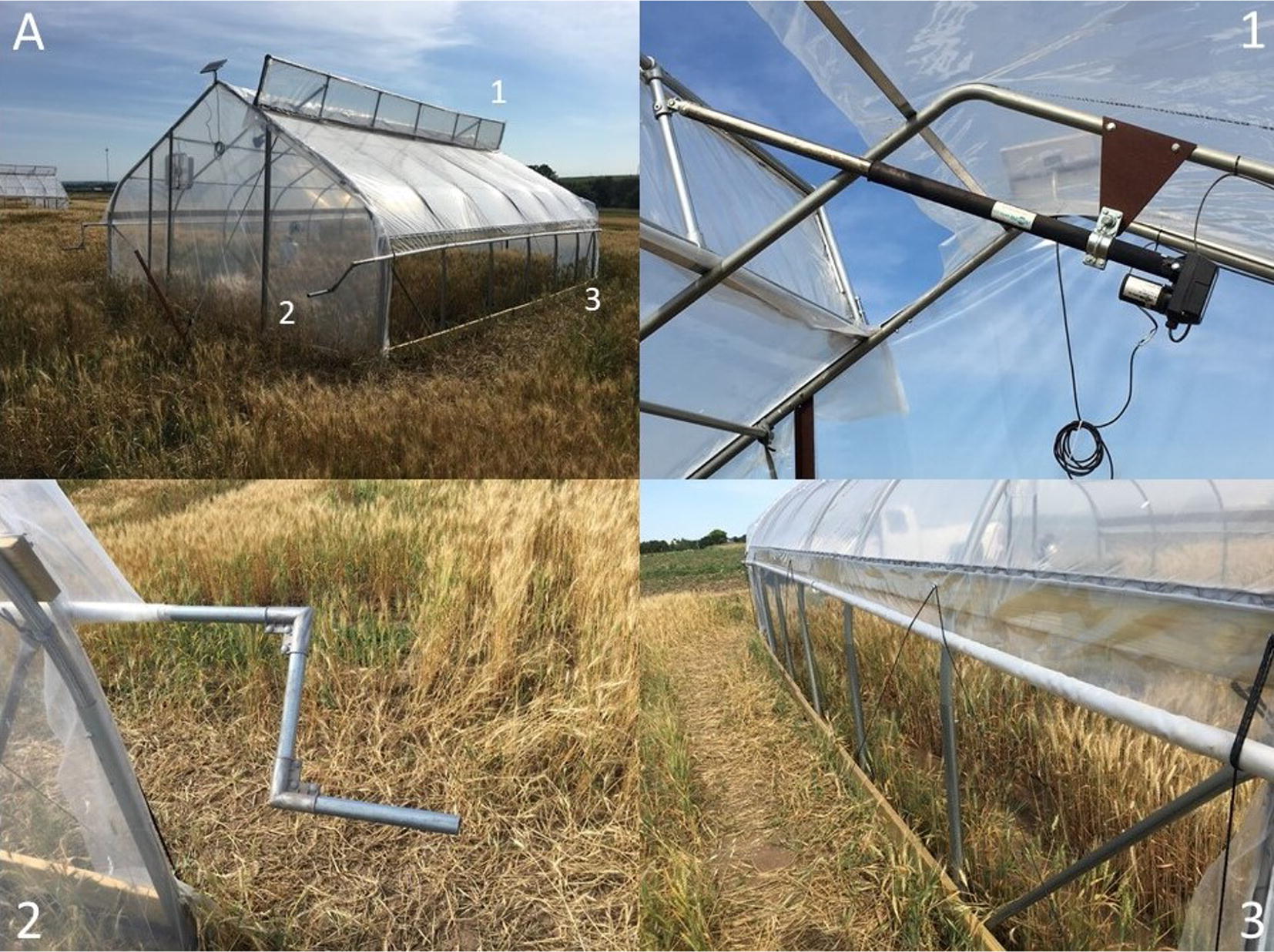
Vent system layout. **A** HNT heat tent during daytime 1: venture manufacturing 12 V linear actuator used to open top vent. 2: Handle used to manually operate side roll up ventilation. 3: Side rolled up with polypropylene rope securing it against the tent

A secondary frame was built that was 0.6 m wide and 7.2 m long from the same material as the structure of the heat tent. The frame was placed at the top of the apex with the bottom hinged to the tent structure. This setup allowed the vent to open up and away from the apex allowing as much heat as possible to escape through the vent (Fig. 1A). Two linear actuator motors (Venture Manufacturing) were attached to the vent framework (Fig. 1.1). When powered, these motors would open and close the vent framework via the hinges that connect the vent to the main structure. The power for these linear actuators was provided by a 12v VRLA battery that was connected to a solar panel attached to the front apex of the roof. The solar panel charged the 12v battery during the day, allowing the battery to be charged and used throughout the experiment. The battery power was run through a thermostat controller (Dayton Temperature Control 4LZ95A) (Fig. 2.1). During the day the thermostat was set to 0 °C to ensure the vent stayed open throughout the day and at night at 44 °C to keep the vent closed throughout the night.

**Fig. 2:**
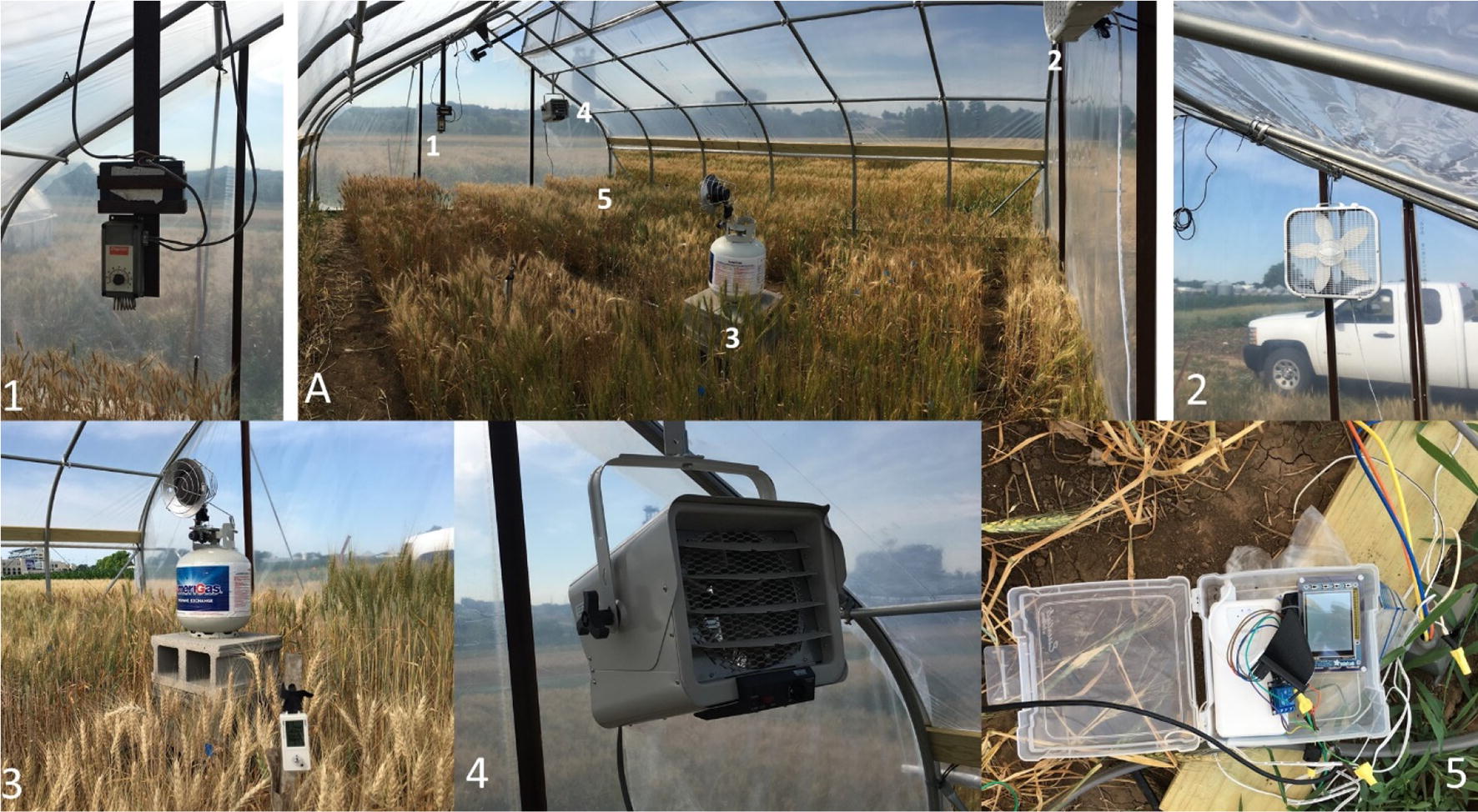
Heating system layout. **A** Layout of heating system within the Tent. 1: Dayton Thermostat Controller used to raise and lower the top vent. 2: Lasko 20 in. Box Fan. 3: Hobo temperature/relative humidity sensor and propane tank with the Sunrite™ by Mr. Heater^®^ 15,000 BTU tank top portable propane heater. 4: Thermosphere 5000-W Ceiling-Mount garage heater. 5: Thermostat Controller System built using a Raspberry Pi

#### Side Roll Vents

The purpose of the side roll vents was to allow for maximum air flow through the wheat canopy during the day. Combined with the top vent, the side roll up vents on both sides of the tent allowed ambient air to flow through the tent and forced hot air to be expelled through the top vent. Pressure treated 2″ × 6″ (5.1 cm × 15.24 cm) wooden boards were installed along the very bottom of the side walls with screws that were rated to attach wood to metal (Everbilt #14 2-3/4 in. Phillips Flat-Head Self-Drilling Screw). The boards used were 3.04 m in length, which required multiple boards to cover the length of the side walls. The boards were attached to each other using deck screws to ensure stability (Deckmate #9 × 3 in. Star Flat-Head Wood Deck Screws). These wooden boards were then run across the side wall at 1.5 m above the base and secured in the same fashion (Fig. 1.3).

The horizontal lock channel and wiggle wire was installed on the upper third of the outside face of the top row of wooden boards with metal to wood screws (Teks #12 1 in. Hex-Head Self-Drilling Screws). The vertical lock channel along the end walls was then installed down along the frame, so the end wall plastic could be secured all the way to the ground. It was at this point during the set up that the new plastic was applied on all the tents. The side walls were done first with enough plastic hanging down from the top row of wooden boards to reach the ground. The plastic was secured along the vertical lock channel on the side walls from the top to the bottom row of wooden boards and then left loose below that.

Eye screws (Everbilt #206 × 1-3/8 in. Zinc-Plated Steel Screw Eye) were installed on both the top and bottom row of boards at either end and then alternating between the top and the bottom set of boards to form a zigzag pattern (Fig. 1.3). The top row of eye screws were placed through the hanging plastic while the bottom row of eye screws did not go through the plastic so that the plastic could be rolled up.

To create the metal bar that the extra plastic would be rolled up on resulting in the side roll vents, three pieces of 3.5 cm × 3.2 m 17-gauge galvanized piping were combined using Teks #12 1 in. Hex-Head Self-Drilling Screws. Two of the pieces were used in full while the third was cut to 1.52 m in length allowing an extra 0.3 m of piping on either end of the heat tent. In total, for each side wall a 7.92 m length of piping was used. Each pole had a tapered end and a full end. The tapered ends of the poles were inserted into the full ends and then screwed together with the Tek screws. The screws were then wrapped in duct tape to ensure the screw heads would not rip the plastic.

A handle was added to one end of the roll up bar to rotate the bar to facilitate the rolling up and lowering of the side walls (Fig. 1.2). The 3.5 cm × 3.2 m 17-gauge galvanized piping was cut into two 0.3 m lengths and then attached to the end using an aluminum gate ell. Two pieces of piping and two aluminum gate ells were used to create the handle for each roll up, on either sides of the tent. The 7.92 m long pipe was then laid along the side walls of the heat tent on top of the excess plastic that was draped on the ground. The plastic was evenly wrapped around the pole in a clockwise manner and duct taped every 1 m to attach the pipe firmly with the plastic.

A piece of polypropylene rope was attached to the top eye screws on the wooden boards on the end with the handle and a loop made on the other end so that it could be attached to a screw on the interior of the tent to hold the roll up when the side walls were open. The handle was then rotated in a clockwise rotation to roll the plastic up to the top row of the wooden boards and then secured with the loop that was previously put in place. The same polypropylene rope was then run from the top eye screw on one end of the top wooden board to a similar screw on the bottom wooden board and then pulled through the eye screws in the zig zag pattern that was made previously. Once the rope had reached the far end, it was run through both the top and bottom eye screws, pulled tight, and secured. This rope was necessary to keep the roll up flush against the heat tent during the rolling process, and also prevented billowing when the side walls were rolled down (Fig. 1.3). The end walls then had their polyethylene film applied over the top of the sidewall plastic so as to seal the ends of the heat tents (Additional file 1: Fig. S1).

#### Heating system

Before any decisions could be made on the size and type of heating system, the amount of heat that was necessary to raise the tent to the targeted temperature was calculated by using the formula $Q = \frac{T*A}{R}$. The amount of heat (Q), British Thermal Unit per hour (BTU h^−1^), required to attain the target temperature differential (ΔT in °F) was figured using the surface area of the heat tent (A in ft^2^) and the capacity of the covering of the heat tent to resist heat flow (R in inch-pound). Some manufacturers or materials may not provide an R value but rather a heat loss value (U) which is equal to 1/R. The heat tents had a surface area of 1100 square feet and an R value of 0.87. The target maximum temperature difference inside the tent from the outside ambient temperature during the night was 4 °C or 7.2 °F. Using these values in the above formula, the minimum heat required to raise temperature inside the tent by 4 °C was 9103 BTU h^−1^ or 2667 W (1 BTU = 0.293 W).

The Thermosphere Ceiling-Mount Garage Heater was installed in the tent hanging from a horizontal structural pipe two-thirds of the distance from the apex (Fig. 2.4). The capacity of this unit was 5000 W, 17,065 BTU h^−1^, 240 V (model number PH-950). In addition to the heater, a single box fan (Lasko Ltd.) was hung in the opposite end of the tents to ensure air within the tent was circulated throughout the night (Fig. 2.2). These fans drew 75 W each and ran off of an 110v circuit, with the power provided by the generator (Additional file 2: Fig. S2).

This experiment had three independent heat tents running overnight powered with a Caterpillar XQ35 Generator which provided 27 kW of power consistently using 8.8 L of diesel per hour. The diesel was stored in a 3785-liter tank with an electrical pump that was battery operated and used to refill the generator (Additional file 2: Fig. S2). The generator was wired to the heaters using Southwire 8/2 AWG UF-B Underground Feeder Cable with Ground and Southwire 10/2 AWG UF-B Underground Feeder Cable with Ground depending on the length of run between the generator and the heater. The box fans were provided power with HDX 16/3 Indoor/Outdoor Extension Cords.

Although the calculations were accurate for the amount of heat needed to raise the temperature of a typical greenhouse, the modifications made to the heat tent structure affected its ability to retain heat. Hence, an additional source of heat was necessary to maintain the target differential. A Sunrite™ by Mr. Heater^®^ 15,000 BTU Tank Top Portable Propane Heater (Fig. 2.3) was added to achieve the target temperature. The propane heater provided 10,000 BTU h^−1^ on low, 12,000 BTU h^−1^ on medium, and 15,000 BTU h^−1^ on the high setting. The propane heater was set to its medium setting which provided a radiant heat source but was not equipped with a forced air component and can potentially pose a fire hazard on the ground level. Hence, the propane tank and heater were placed on a stand built with cinderblocks to raise it above the height of the wheat and placed directly below the path of the air blown by the box fans. The propane tank top heater increased the interior temperature towards the target temperature via radiant heating and air movement by the fan while the final target differential of 4 °C was achieved and regulated by the electric heater by turning on and off as needed.

A low-level fire hazard did exist with the use of a diesel generator and propane tank top heater. However, the diesel generator itself did not create a fire risk unless a complete component failure occurred. The generator was self-contained on a trailer and had adequate insulation and protective measures to minimize risk. On the other hand, the fire hazard posed by the propane tank can be completely eliminated by increasing the wattage of the original electric heater and eliminate the need for a propane tank top heater.

Another aspect related to utilizing a propane tank top heater is the possibility of CO_2_ build up within the tent and its effects on the plants. Direct estimation of CO_2_ concentration using at least two sensors within each tent would have been an ideal approach to ensure that there were no unintended effects of elevated CO_2_ on the plants. Higher levels of CO_2_ would warrant the addition of more ventilation to allow for fresh air to enter the tents and a ducted ventilation tube for the gasses produced during the combustion of propane. However, no additional ventilation was required for the heat tents as they were not airtight and allowed for ample ventilation. The top vent did not seal when closed and the side roll ups were taped shut on the end walls but were not sealed along the side walls. This inherent ventilation in the design allowed for a continuous flow of fresh air and created the necessity for an extra heat source. This is evident with the increase in BTUs required to raise the interior temperature by 4 °C compared to the exterior. In a completely sealed environment with the same volume as the heat tent, it would only take 8854.4 BTUs to achieve the target temperature and overcome conductive heat loss. However, our system used over 29,000 BTUs which correlates to over 20,000 BTUs being needed to overcome perimeter heat loss and air infiltration heat loss. At that rate of heating, the tent had to complete an air exchange every 1.32 min. While CO_2_ was not directly measured, the combination of frequent air exchanges i.e., the top vent not being sealed which allowed for the warm CO_2_ to escape, and the side roll vents not being sealed which allowed the CO_2_ to escape when cooled would have prevented any excess CO_2_ accumulating within the tent and compounding the effects of the HNT stress.

### Temperature controller system

#### Overall description/functionality

A cyber-physical system is a physical mechanism controlled by computer-based algorithms in real time. This cyber-physical system was designed to monitor the temperature from the outside environment and regulate temperature within the tent. When the temperature inside the tent was not warmer than the outside by 4 °C, the system turned the heater on to help increase or maintain the indoor temperature differential. Otherwise, the heater was turned off and the temperature was continued to be monitored.

#### Design philosophy

This system was designed around a simple, plug-and-play philosophy using a Raspberry Pi, a low-cost, high-performance computer system developed by the Raspberry Pi Foundation [26]. When the system received power, it booted up and began monitoring the outside and inside temperatures. If the system failed to start, which only occurred twice during the HNT stress period, then the faults were isolated into two categories: Raspberry Pi failures and sensor failures. The Raspberry Pi failures were manually tested by checking for sufficient power source (5 V, 2.1A) and verifying the integrity of the microSD card. Sensor failures were detected by checking the power, electrical ground, and data connections to the Raspberry Pi. The system’s simplicity was exhibited in both hardware and software. The system could be separated into its material components rather simply; the Raspberry Pi, solid-state relay, sensors, and 240 V relay could be isolated by disconnecting at most five wires and could be improved and modified easily without affecting the other components. Software could be modified very rapidly through the Python script (Additional file 3) and uploaded to the Raspberry Pi within minutes by modifying the microSD card.

#### Hardware components and connections

**Fig. 3:**
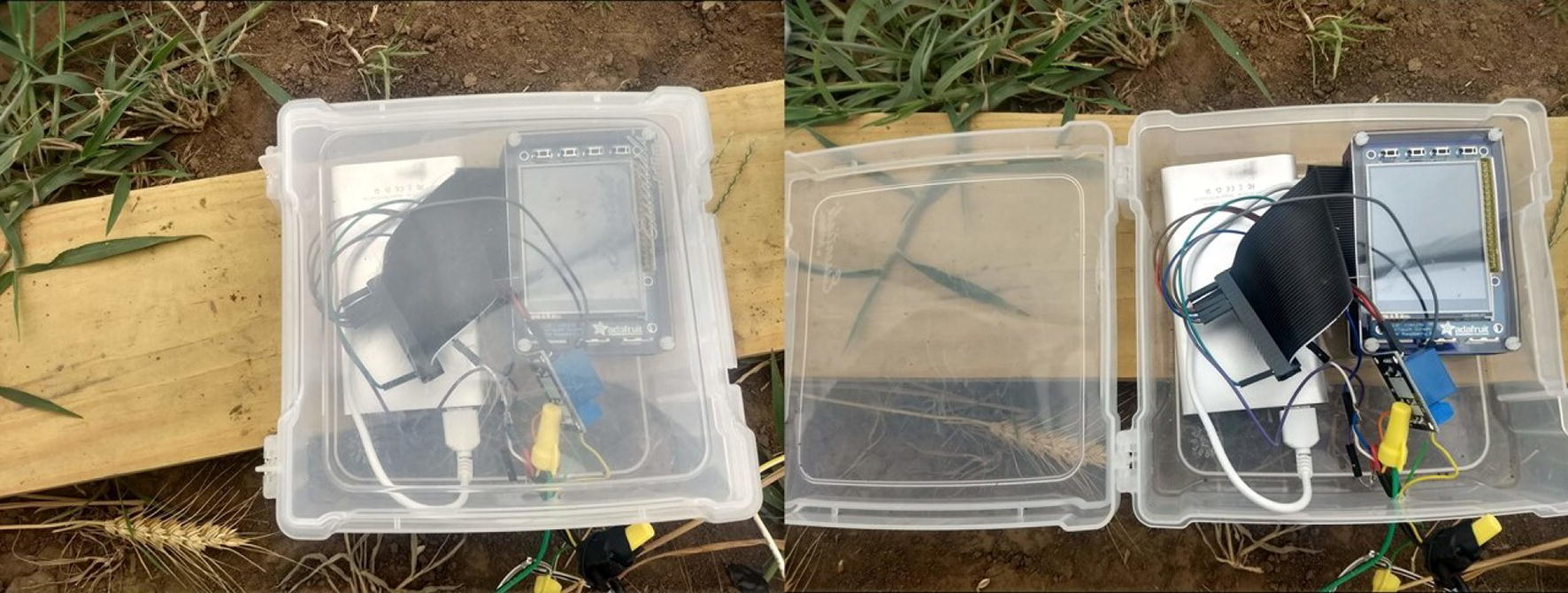
Waterproof enclosure for Raspberry Pi and electrical system. The system was contained within a plastic box that latched closed (left) to protect the underlying circuitry and opened (right) to allow access to the system. Inside each enclosure was a battery pack, USB to microUSB cable to supply power, one Raspberry Pi computer with touchscreen display, a ribbon cable to extend connections to the computer, and a blue solid-state relay. A hole was drilled in the side of the enclosure to facilitate electrical connections to the heater circuit; this hole was filled with caulk for water protection

The thermostat system consisted of several hardware components: a Raspberry Pi, solid-state relay, 24VAC adapter, 240 V relay, and two DS18B20 temperature sensors. Additionally, the system was placed within a plastic housing for water- and dust-proofing (Fig. 3). The Raspberry Pi was connected to the solid-state relay by three wires: 5 V power, electrical ground, and a signal wire. A high bit on the signal wire forced the relay to complete the connection to the heater. The following pin assignments were based on the physical numbering scheme on the Raspberry Pi Model 3B:

- The 5 V connection was routed to pin 2.
- The ground connection was routed to pin 9.
- The signal connection was routed to pin 11.

**Fig. 4:**
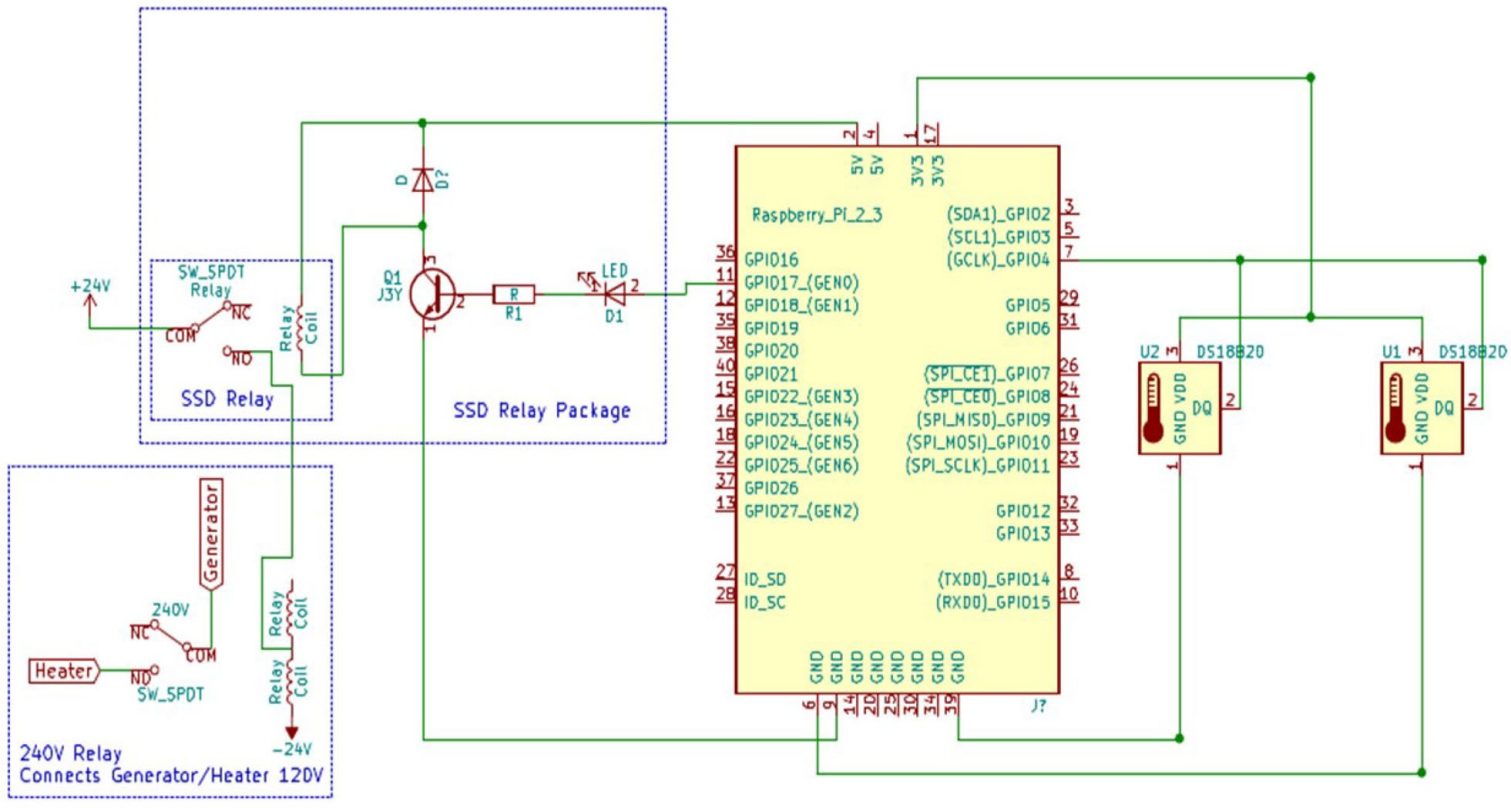
System wiring diagram

The solid-state relay was connected to the 240 V relay and 24VAC adapter. This relay caused the other relay to engage and helped complete the circuit to the heater, as the single relay itself could not support the heater’s electrical load. Two ports from the solid-state relay were used: common and normally open (NO), which were chosen for safety because the heater circuit would not normally be electrically active. The common lead was connected to one lead of the 24VAC adapter, and the NO lead was connected directly to the 24VAC lead of the 240 V relay. In this manner, the solid-state relay completed a circuit between the 24VAC adapter and the 240 V relay (Fig. 4).

The 24VAC adapter was connected to power via the generator cables. The adapter provided power to the 240 V relay and heater circuit. An unpolarized electrical plug was attached to the input terminals. Electrical wire (14-gauge) was connected to each terminal of the plug and then connected to the generator lines; the ground lead was connected to the generator ground, and the power lead was connected to the black 120 V line of the generator. The 240 V relay had four connections: two inputs and two outputs to the heater. One input has been described above and was directly connected to the NO lead of the solid-state relay. The common input terminal was connected directly to the other terminal of the 24VAC adapter. The common output terminal was wired to one of the generator’s 120 V lines, and the NO terminal was connected to the corresponding line on the heater. The neutral and second 120 V lines were connected directly from the generator to the heater; the relay switched a single 120 V line to complete the circuit (Fig. 4).

The two DS18B20 temperature sensors were wired in parallel and shared the same three pin connections. A 4.7 kΩ pull-up resistor was connected between the power and data lines and prevented a floating wire state and a wire short [27]. The following pin assignments were similar to the solid-state relay:

- The 3.3 V connection was routed to pin 1.
- The ground connection was split and routed to pins 6 and 39.
- The data connection was routed to pin 7.

#### Software description

The software was written in a Python script, version 2.7 (Additional file 3) [28]. This allowed for rapid prototyping and quick implementation of the sensor readings. When the Raspberry Pi was booted, the software first polled the system bus for the sensors and added them to a list, which allowed for more sensors to be connected to the system. Next, the signal pin of the solid-state relay was set-up via software for toggling: otherwise, the pin would either be on or off. Then, the data log file was opened and a blank line was appended to delimit the start of a new session of logging. This log file was in comma separated value format for easy importing to Microsoft Excel or any other spreadsheet program.

After the setup was completed, the software entered its main loop. First, it attempted to read the sensors that are connected to it using manufacturer code [29]. If the software detected an invalid sensor reading, the error was displayed once the interface was initialized. If the sensor readings were valid, the differential of the indoor and outdoor temperatures was measured and the heater was either turned on or off depending on the value; a value below 4 °C caused the heater to be turned on, and being above 4 °C turned the heater off. Then, the interface was created and updated to the new indoor and outdoor temperatures, as well as the status of the heater (Additional file 4: Fig. S3). If an error occurred with the sensors in the previous steps, then the heater displayed the word “SENSOR” and the connections from the Pi to each sensor was manually verified.

If the elapsed time reached the logging interval, then the current time, indoor and outdoor temperatures, and the heater’s status were recorded to file. If the amount of time elapsed had not reached the interval, a nested loop was executed. The system would go into a sleep mode for half a second and the process was repeated until the target interval had reached. Once the interval had been reached and the status was recorded, the next loop iteration would commence.

### Crop cultivation

A field experiment was conducted at the Agronomy research farm at Manhattan (39°11′N, 96°35′W), Kansas. In this experiment, five prominent varieties of Kansas (Everest, Larry, SY-Monument, WB 4458, and WB-Cedar) and five breeding lines (Jagger X060724, KS070736 K-1, KS070729 K-26, KS070717 M-1, and P1 X060725) and two exotic genotypes (Tascosa and Tx86A5606) known for differential heat stress response during grain filling [8, 30], were used to study the impact of post-flowering HNT stress under field condition. Wheat genotypes were planted using a tractor and research plot grain drill with global positioning system (GPS) guidance system on 17th October 2018. Each replicate plot per genotype comprised of six rows with each row being 4-m long (6 rows occupied 1.15 m, with each row placed 0.19 m apart). The plots were top dressed with 45 kg N ha^−1^ (Urea ammonium nitrate solution) on 17th February 2018. Both the control and the stress plots were irrigated throughout the experiment, even during the HNT stress period, either through rainfall or manually once every week to avoid confounded by water-deficit stress. Days to complete flowering across the twelve genotypes was not more than 5 days. HNT treatment was imposed during grain filling using the custom designed heat tents. Twelve winter wheat genotypes were successfully exposed to an average night time differential of + 3.2 °C (interior; inside heat tents) during the grain filling (10 days after 50% flowering to physiological maturity), compared to ambient night-time temperature (exterior; outside heat tents).

### Biological data collection

#### Chlorophyll fluorescence

Five representative plants for each genotype per replicate were randomly selected and tagged at flowering for measuring flag leaf and the main spike chlorophyll fluorescence (Chl-F) in both interior and exterior conditions. Chl-F data was recorded between 1000 and 1300 h by using a portable hand-held fluorometer (FluorPen FP 100, Photon System Instruments, Ltd., Brno, Czech Republic), which gives the effective quantum yield of PSII (QY). Saturating light [intensity approximately 3000 µmol (photons) m^−2^ s^−1^] and measuring light [intensity approximately 0.09 µmol (photons) m^−2^ s^−1^] were used to measure both maximal fluorescence yield (FM′) and actual fluorescence yield (Ft) of light adapted samples, respectively. Subsequently, the effective quantum yield of PSII (QY) was calculated using the formula $QY = \left( {FM^{{\prime }} - Ft} \right)/FM^{{\prime }} = \Delta F/FM^{{\prime }}$ [31]. Electron transport rate (ETR) which indicated the capacity of overall photosynthesis was calculated by using the formula as described previously [31].

$$ ETR = QY \times PAR \times 0.84 \times 0.5 $$

where QY is the effective quantum yield of PSII, PAR is actual photosynthetic active radiation (µmol (photons) m^−2^ s^−1^), 0.84 is an approximate level of light being absorbed by the leaf, and 0.5 is the ratio of PSII to PSI reaction centers. Three measurements were taken along the middle of the flag leaf blade and spikes on each replicate plant and averaged.

#### Grain yield

At physiological maturity (Zadoks growth scale 9-ripening; not dented by thumbnail), replicates of 1-m row length from four central rows was manually cut in each plot to minimize border effects. Spikes were separated from the stem and dried for 96 h at 40 °C and spikes were threshed using an LD 180 Laboratory thresher (Wintersteiger, Ried im Innkreis, Austria) and grain yield was recorded.

### Statistical analysis

The experiment was conducted in a split-plot randomized complete block design with temperature as the main plot factor and genotype as the sub-plot factor. Replicated observations for each trait were analyzed for means and standard errors. ANOVA was performed using GenStat [32].

## Results and discussion

**Fig. 5:**
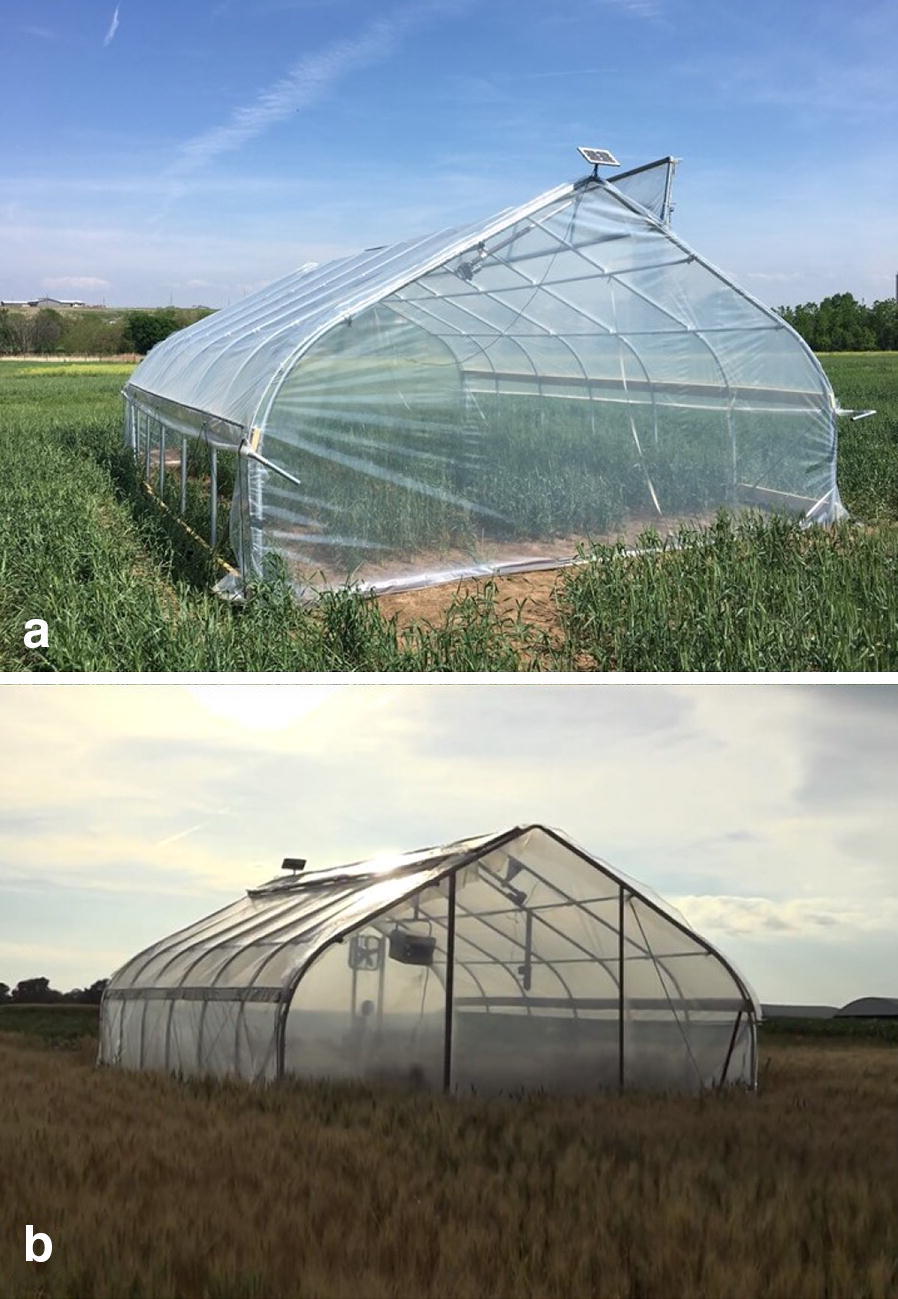
Day setting versus night setting. **a** Heat Tent in day-time setting with top vent and side wall vents opened up. **b** Heat tent during night-time when heat stress was imposed with the top vent and side wall vents closed

To induce heat stress using the components described above, the process of converting the structures from its day-time setting to its night-time setting began at 7:15 PM every night. A single side wall from each tent was lowered and sealed using duct tape. Alternatively, this could also be accomplished by running a strip of Velcro along the end wall and adhering it to the sidewall plastic. Following the sidewall roll down, the top vent was closed to seal the roof. After all the tents had a single sidewall down and the overhead vents lowered and sealed, the portable power packs were plugged into the Pis to start the systems, to initiate the temperature monitoring programs. Then the generator was turned on to supply power to each tent. The Pi system was considered operational if the electric heater was running with the red indicator light. The additional propane heater was turned on after all the other parts of the system were fully operational. As a final step the second side wall was lowered and sealed to fully enclose the tent for the night (Fig. 5b).

At 5:45 AM every morning, the generator was shut down, so that no electricity was flowing through the system. The sidewalls were unsealed from the end walls, rolled up, and secured at the top with polypropylene rope, the propane heater was shut down, the top vent opened (Fig. 5a), and the battery from the Pi system was removed to shut it down for the day. The batteries were removed every day but only recharged every other day off site from the experiment. The propane tanks were refilled after three consecutive nights of HNT stress.

The system was monitored through a combination of sensors in the interior of the tent and the exterior. One HOBO UX 100-011 temperature/relative humidity data loggers (Onset Computer Corp., Bourne, MA) with a sensitivity of 0.2 °C was placed in a central location on the experimental plot to log the ambient air temperature and humidity. Similarly, two HOBO sensors were placed within each tent to log both day-time and night-time temperature and humidity. The Pi temperature sensing and controller system was also equipped with one sensor inside the tent and the other sensor placed outside each tent having an accuracy of 0.5 °C. In total, each tent was equipped with three sensors. The two main goals of this field set up was to induce a HNT stress with a pre-decided target differential supported by the Pi’s programming, and to ensure an even distribution of the heat throughout the night to minimize a temperature gradient or irregular warming patterns within the tent. In addition, the aim during the day-time was to ensure temperatures within the tent were close to the outside ambient temperature.

### Distribution of heat

To ensure that the tent was not experiencing a gradient in temperature within the tent, two different HOBO sensors were placed within the wheat plots on opposite sides of the tents directly above the canopy to measure the temperature throughout the night and day at 15-min interval. The distribution of heat was enabled through the box fan that operated from one end and the electric heater that ran on the opposite side. The electric heater with an inbuilt forced air system complemented the box fan on the other end to distribute the heat evenly throughout the tent.

**Fig. 6:**
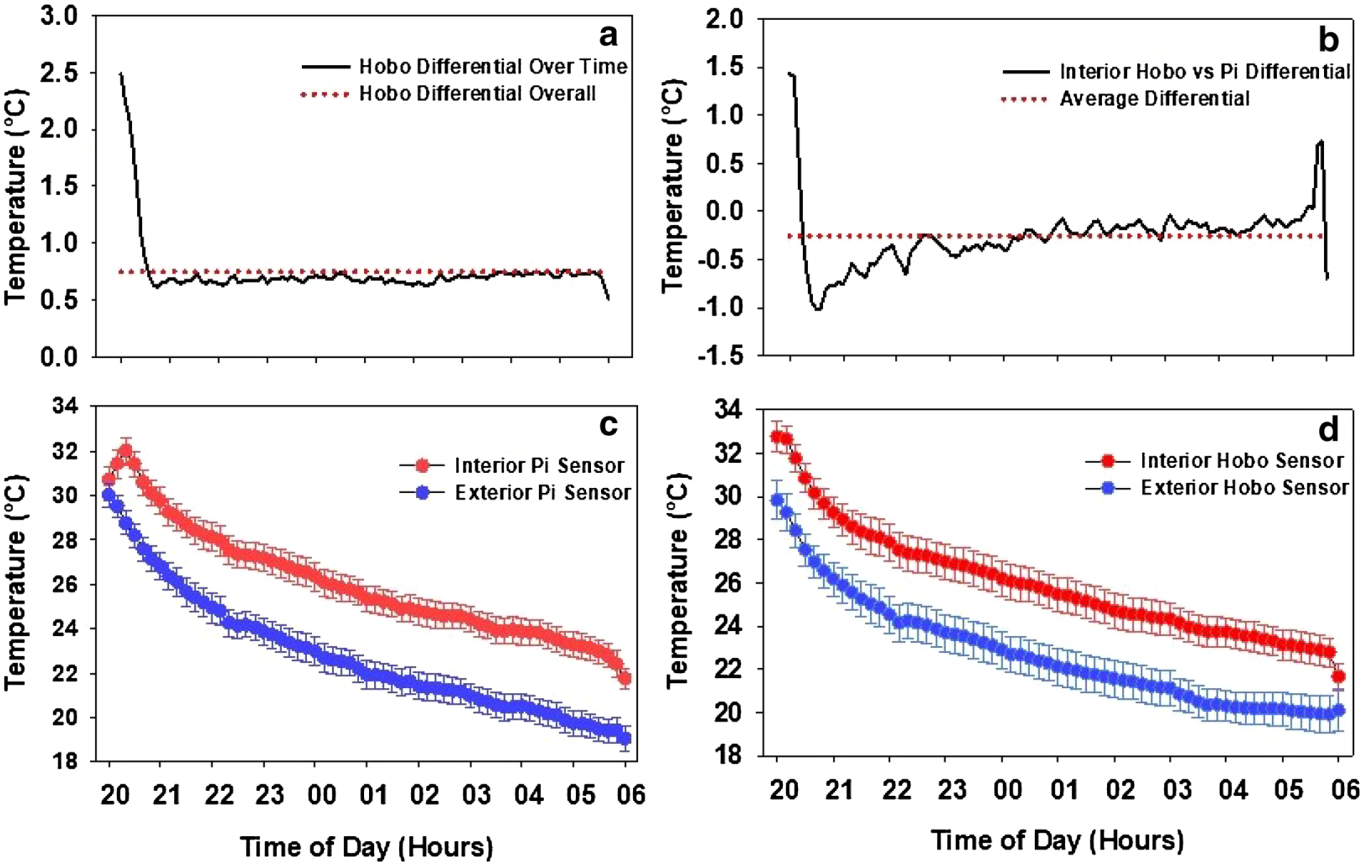
Temperature comparison between sensors. **a** HOBO versus HOBO HNT differential within the same tent, **b** Interior HOBO versus Interior Pi temperature differential, **c** Interior Pi versus Exterior Pi temperature during HNT stress, **d** Interior HOBO versus Exterior HOBO temperature during HNT stress

The difference between the two HOBO sensors within the tent was on average 0.75 °C (Fig. 6a). The HOBO sensors at the start of the treatment recorded a large differential of 2.5 °C on average due to the heating system turning on to bring the tent up to its target differential temperature and possibly due to one of the sensors placed in the path of the heater’s air flow. Once the tents reached the target temperature (roughly around 9 PM) the difference between the two HOBO temperature loggers leveled out and were within the range of 0.5 and 0.75 °C. In addition, the distribution of heat was also confirmed by comparing the average of two HOBO temperature readings with the interior Pi system sensor. Overall average difference between the HOBO sensors and the Pi sensors was -0.25 °C, with the Pi system sensors reading 0.25 °C warmer than the HOBOs (Fig. 6b). A consistent but small temperature difference was recorded within the tent indicating even distribution of heat.

### Temperature differential

The second goal of the heat tent system was to maintain a set temperature differential between the interior of the heat tent and the exterior. The tents were programmed to maintain a temperature differential of 4 °C throughout the night. Comparing the Pi systems sensors, the tents were able to maintain an average differential of 3.2 °C consistently throughout the heat stress period (Fig. 6c). The figure shows that the temperature at 8:00 PM were almost equal at the time the tents were sealed and the heating system was turned on. An hour after the start, the temperature reached a stable differential and then followed the exterior temperature throughout the night, while still maintaining the differential.

This effect can also be seen in Fig. 6d which is a comparison between the temperature recorded from HOBO sensors placed within and outside the heat tent. The elevated interior temperature follows the exterior temperature through the night and in the morning both outside and the inside tent temperatures return to the same level, after the tents are opened. The HOBO sensors also measured an average of 3.2 °C temperature differential throughout the experiment, providing additional independent validation of the system’s successful imposition of HNT stress.

### Ambient day time temperature and relative humidity

The main concern during the day for the heat tent infrastructure was its ability to regulate the air temperature inside the tent, so that the wheat inside the tent is exposed to similar conditions as outside the tent. The readings from both HOBO data loggers inside each tent were averaged and on comparing to the exterior HOBO indicated 0.8 °C warmer temperature within the tent during the day.

**Fig. 7:**
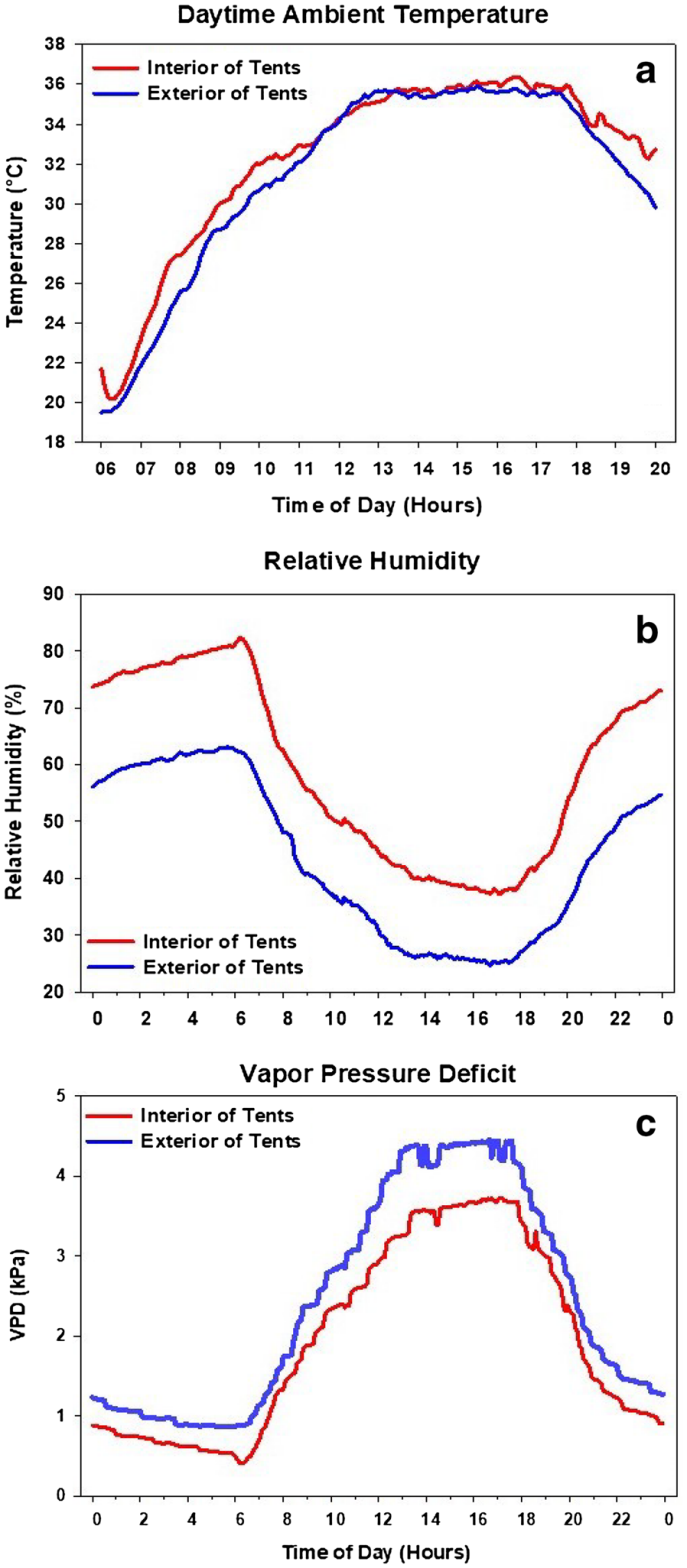
Ambient temperature and relative humidity comparison. **a** Day-time ambient temperature comparison between the interior HOBOs and the exterior HOBO. **b** The average relative humidity of the interior of the tent HOBOs compared to the exterior HOBO. **c** Comparison of the Vapor Pressure Deficit between the interior and exterior of the heat tents

The interior temperature of the tents warmed quicker in the morning than the exterior temperature (Fig. 7a). This rise in temperature compared to the ambient temperature can be credited to the greenhouse effect from the plastic on the heat tents and the typical lack of air movement in the morning hours. With low air movement there is less pressure differential between the inside and outside of the top vent, resulting in much slower circulation of air out of the tent. This effect caused the interior temperature of the tents to reach a maximum of 2.54 °C higher than the exterior by 7:40 AM, with both becoming equal by 12:05 PM after which the average exterior temperature was higher than the interior temperature. The temperatures stayed almost equal from noon until 6:30 PM. After 6:30 PM the temperature differential between the inside of the tents compared to the exterior rose until the heat stress began. The rise in temperature in the later hours of the day can be attributed to the tent retaining the day’s heat longer due to its covering versus the open exterior.

On average, the tent’s relative humidity was 15.6% higher than the ambient average (Fig. 7b). The difference between the interior and exterior peaked towards the end of the HNT stress exposure at 6:00 AM and then reduced throughout the morning until noon. After noon, there was a consistently higher level of humidity inside the tent until 6:00 PM in which the difference receded until the stress imposition began again. It is also apparent through the data that the relative humidity differential between the interior and the exterior was the greatest during the HNT stress period when the tent was sealed. Using the relative humidity and air temperature data from inside and outside of the heat tents, the vapor pressure difference (VPD) was calculated through both the stress and non-stress periods. The VPD was highest during the day when the temperature was at its warmest and the relative humidity at the lowest (Fig. 7c). To account for any variation in evaporation and transpiration due to the changes in RH and VPD within the tents, the plots were irrigated weekly from flowering until harvest.

### Physiological and yield response to HNT

**Fig. 8:**
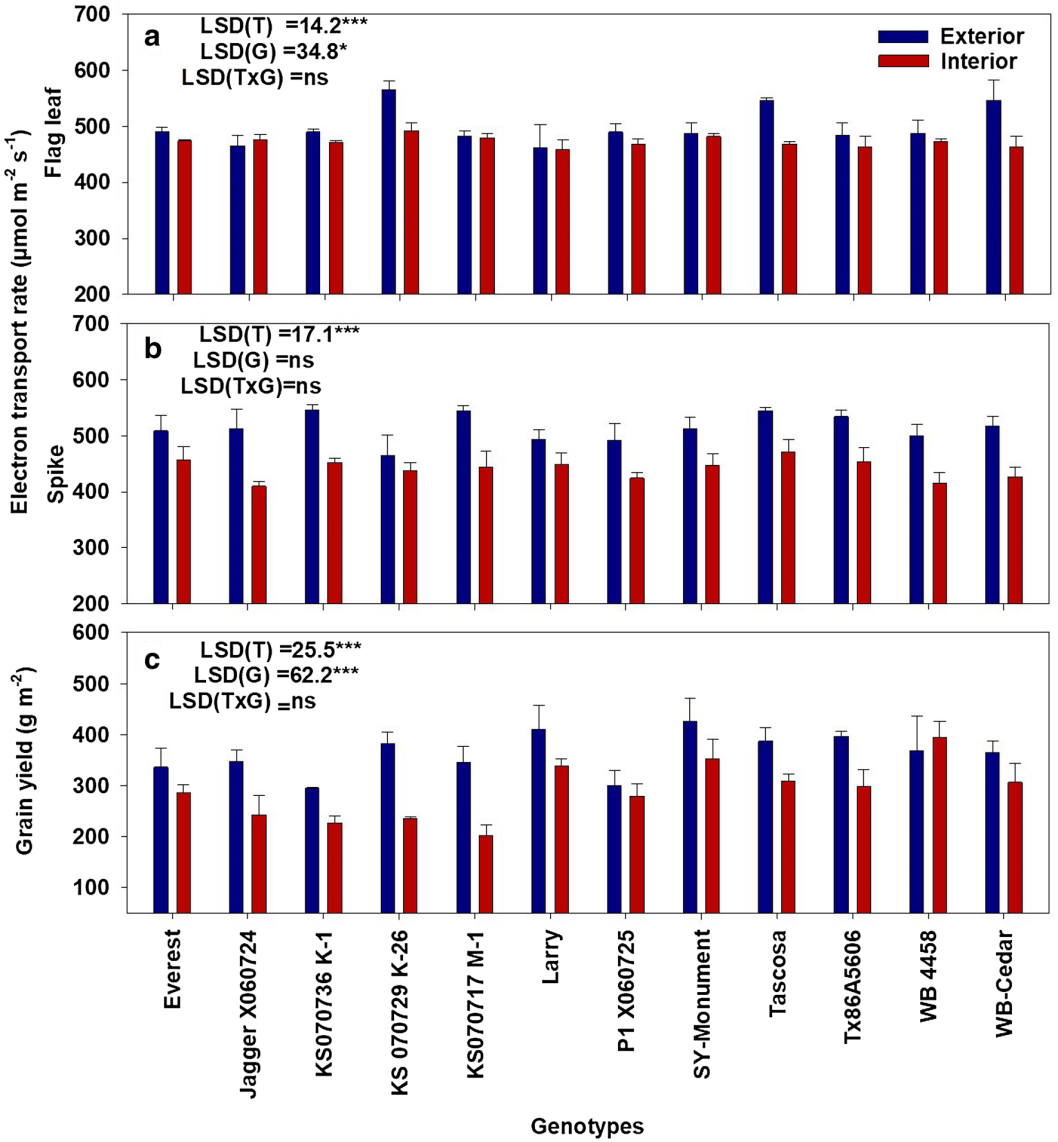
Physiological and yield response to HNT. Flag leaf (**a**) and spike (**b**) electron transport rate recorded 7 days after treatment imposition and grain yield (**c**) of twelve winter wheat genotypes under exterior (control) and interior (HNT treatment) conditions. Analysis of variance with least significant difference (LSD) is presented for each trait. *T* treatment, *G* genotype, *ns* non-significant. *P < 0.05; ***P < 0.001. Bars indicate mean  ±  standard error (n  =  3)

A significant (P < 0.001) decline in the electron transport rate (ETR) of the flag leaves was observed after seven days of treatment imposition (Fig. 8a). Among the tested genotypes, KS070717 M-1 and Larry recorded the lowest percent reduction (< 1%) in flag leaf ETR under heat stress compared to control, whereas Tascosa (14.3%) followed by KS 070729 K-26 (13%) recorded the highest reduction in flag leaf ETR (Fig. 8a). Similarly, a significant (P < 0.001) treatment impact was recorded for main spike ETR, ranging from 5.7% (KS 070729 K-26) to 19.4% (KS070717 M-1) with HNT compared to control, with an average reduction of 14.3% (Fig. 8b). Significant (P < 0.001) effect of temperature and genotype were observed with grain yield but with no treatment and genotype interaction (Fig. 8c). Eleven genotypes (except WB 4458) out of the twelve responded to heat stress treatment by reducing their grain yield, with an average reduction of 20.3%, ranging between 6.9% in P1 X060725 and 41.4% in KS070717 M-1 (Fig. 8c). Under HNT stress exposure during grain-filling (Fig. 8c), WB 4458 had the highest grain yield (394.2 g m^2^) followed by SY-Monument (352.5 g m^2^), whereas the lowest grain yield was recorded in KS070717 M-1 (202.4 g m^2^).

### System improvements

By further improving, the system can be adequately scaled up for phenotyping larger genetic diversity and the gap between the target average temperature differential (4 °C) and the achieved (3.2 °C) can be narrowed through minor improvements to the system.

1. Adding more temperature sensors will help obtain an average temperature from multiple points within the tent which will lead to improved heating accuracy. The total number of sensors that can be attached to an individual Pi is 117 which allows ample capacity for a single Raspberry Pi to handle a much larger and extensive setup [33]. Additional sensors that sense relative humidity, CO_2_ and light intensity will track microclimatic parameters within the tent and facilitate in maintaining target experimental conditions.
2. Adding another fan can improve uniformity in distribution of heat within the tent. This will help the extra sensors accurately determine the temperature within the tent and improve the system’s capabilities when designing a larger experiment.
3. Higher precision sensors—The sensors that were used within the system connected to the Pi had an accuracy of 0.5 °C. Sensors with higher accuracy will result in less variable temperature readings and when averaged with the additional sensors throughout the tent a much more precise reading of the temperature can be attained.
4. Increasing the recording frequency in the Pi system. This will help by turning the heater on and off as frequently as necessary. The changes made to the tents to help maintain ambient air temperature during the day adds to the heat loss during night. The longer amount of time between readings from the Pi system results in a larger swing in temperature while the heater is off. With more frequent readings, the heater would be able to modulate the temperature more efficiently.
5. Heater that receives input air from the exterior via venting—This will help mitigate the increased relative humidity and possible buildup of CO_2_ within the tent. This would allow fresh air with an ambient level of relative humidity and CO_2_ to enter the system and be circulated throughout the tent instead of the same air from within the tent being drawn into the heater and then dispersed.

## Conclusions

A robust field-based system with the use of roll up and down side ventilation, top ventilation, a heating system, and a cyber-physical system using a Raspberry Pi was constructed that was able to effectively impose HNT stress while automatically following the dynamic changes of the outside environment. The top and side ventilation also allowed the system to maintain near ambient temperatures throughout the day without having to physically remove the tent from the field, while still being able to seal them overnight providing a HNT stress exposure on multiple wheat genotypes in a field setting. The system and the methodology followed indicated that crop agronomic and physiological responses to HNT can be effectively captured under realistic field conditions to help ongoing breeding efforts aimed at improving crops adaptation to changing climates. This system can be altered, improved based on some of the above recommendations. Although the methodology has only been tested on wheat, since it is not reliant on access to any hardwired utilities and is reliable, simple, and cost-effective (see list of the parts and cost per tent in Additional file 5), this system can be used to phenotype other crops or plants for HNT responses.

## Additional files

**Additional file 1: Fig. S1.** Heat tent before and after end wall plastic application.

**Additional file 2: Fig. S2.** Caterpillar XQ35 Generator and 3785-l diesel tank.

**Additional file 3.** Thermostat Controller Python Script.

**Additional file 4: Fig. S3.** Raspberry Pi displaying interior temperature, exterior temperature, and status of the heater.

**Additional file 5.** Parts list and cost per tent.

## Abbreviations

HNT: high night-time temperature
HDT: high day-time temperature
NO: normally open
VPD: vapor pressure deficit
RH: relative humidity
