# Supplementary figures and images for: Integrating field-based heat tents and cyber-physical system technology to phenotype high night-time temperature impact on winter wheat
Source: Plant Methods. 2019 Apr 24;15:41. doi: 10.1186/s13007-019-0424-x (PMC6480702; doi:10.1186/s13007-019-0424-x)

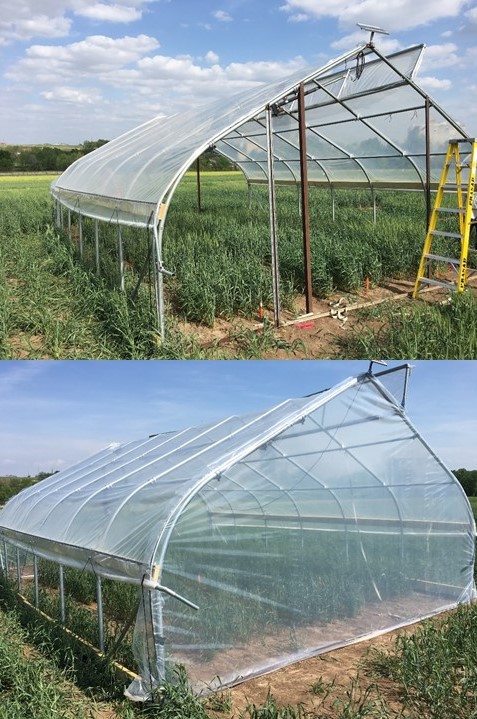

Supplement: Supplementary file 1 — Additional file 1: Fig. S1. Heat tent before and after end wall plastic application. [file 13007_2019_424_MOESM1_ESM.jpg]

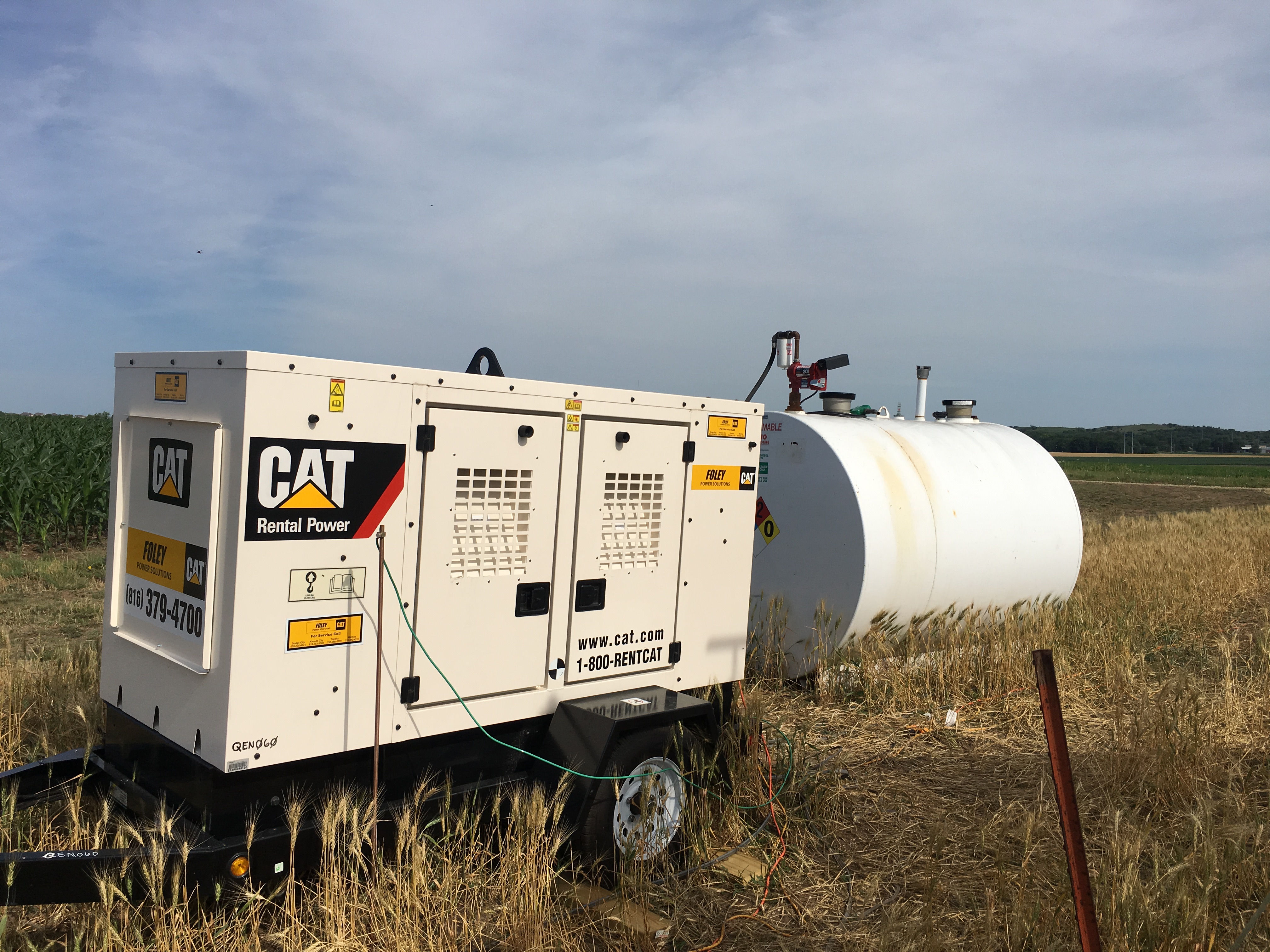

Supplement: Supplementary file 2 — Additional file 2: Fig. S2. Caterpillar XQ35 Generator and 3785-l diesel tank. [file 13007_2019_424_MOESM2_ESM.jpg]

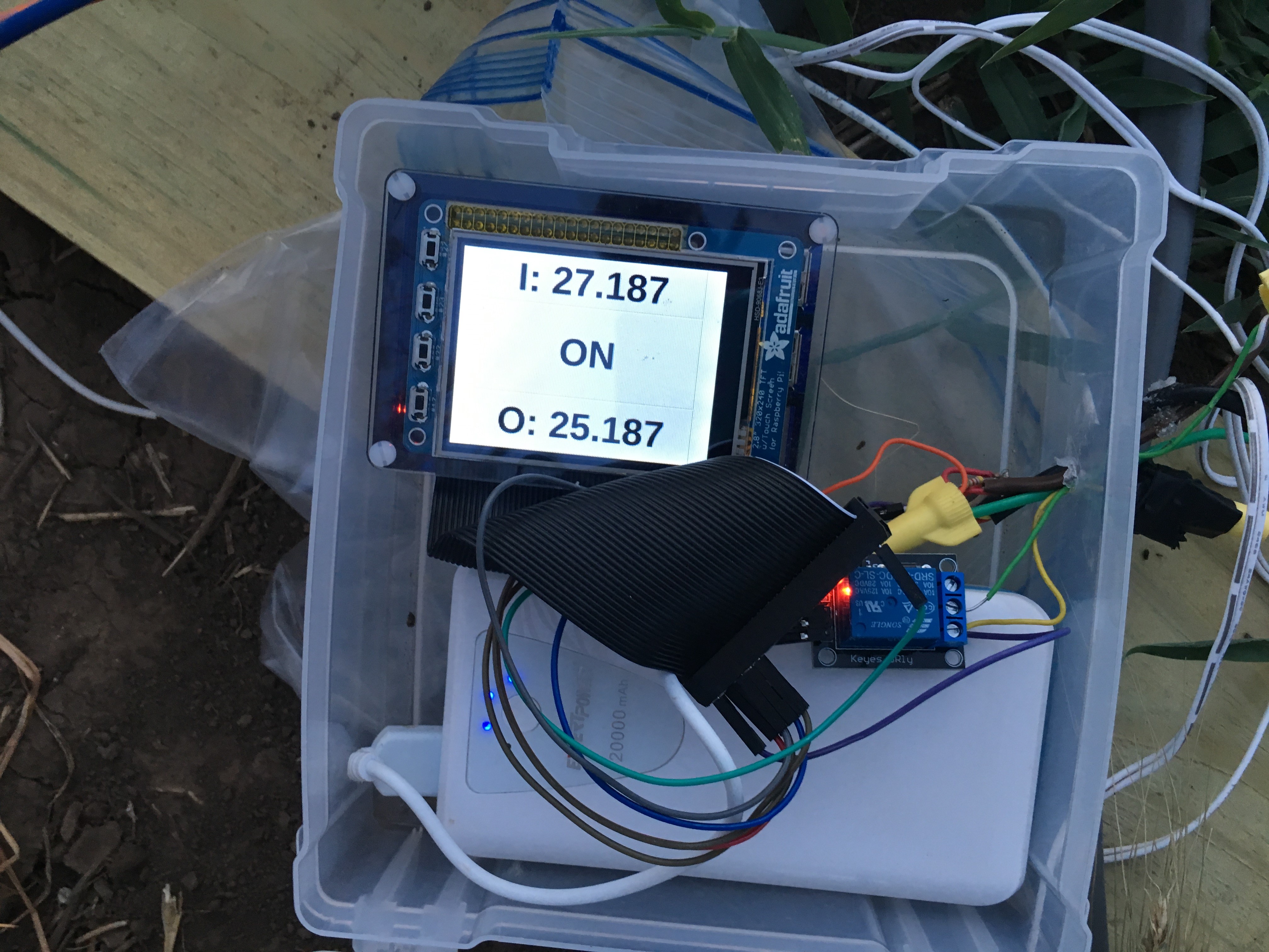

Supplement: Supplementary file 4 — Additional file 4: Fig. S3. Raspberry Pi displaying interior temperature, exterior temperature, and status of the heater. [file 13007_2019_424_MOESM4_ESM.jpg]
